# Progressive multiple sclerosis, cognitive function, and quality of life

**DOI:** 10.1002/brb3.875

**Published:** 2018-01-05

**Authors:** Helene Højsgaard Chow, Karen Schreiber, Melinda Magyari, Cecilie Ammitzbøll, Lars Börnsen, Jeppe Romme Christensen, Rikke Ratzer, Per Soelberg Sørensen, Finn Sellebjerg

**Affiliations:** ^1^ Department of Neurology Danish Multiple Sclerosis Center Rigshospitalet University of Copenhagen Copenhagen Denmark

**Keywords:** cognitive function, multiple sclerosis, physical function, progressive multiple sclerosis, quality of life

## Abstract

**Background:**

Patients with progressive multiple sclerosis (MS) often have cognitive impairment in addition to physical impairment. The burden of cognitive and physical impairment progresses over time, and may be major determinants of quality of life. The aim of this study was to assess to which degree quality of life correlates with physical and cognitive function in progressive MS.

**Methods:**

This is a retrospective study of 52 patients with primary progressive (*N *= 18) and secondary progressive MS (*N *= 34). Physical disability was assessed using the Expanded Disability Status Scale, Timed 25 Foot Walk (T25FW) test and 9‐Hole Peg Test (9HPT). Cognitive function was assessed using Symbol Digit Modalities Test (SDMT), Paced Auditory Serial Addition Test, and Trail Making Test B (TRAIL‐B). In addition, quality of life was assessed by the Short Form 36 (SF‐36) questionnaire.

**Results:**

Only measures of cognitive function correlated with the overall SF‐36 quality of life score and the Mental Component Summary score from the SF‐36. The only physical measure that correlated with a measure of quality of life was T25FW test, which correlated with the Physical Component Summary from the SF‐36. We found no other significant correlations between the measures of cognitive function and the overall physical measures but interestingly, we found a possible relationship between the 9HPT score for the nondominant hand and the SDMT and TRAIL‐B.

**Conclusion:**

Our findings support inclusion of measures of cognitive function in the assessment of patients with progressive MS as these correlated closer with quality of life than measures of physical impairment.

## 1 BACKGROUND

The progressive forms of multiple sclerosis (MS) are characterized by the accumulation of neurological disability independent of relapses. Progressive MS is divided into primary progressive (PP) MS (approximately 15% of all patients) and secondary progressive (SP) MS that follows a period of relapsing‐remitting (RR) disease course (Lublin et al., 2014; Ontaneda, Fox, & Chataway, 2015). In both cases, progression starts at a mean age of around 40 years. Physical manifestations include motor, sensory, visual, and autonomic symptoms (Compston & Coles, 2008), but many MS patients also experience cognitive problems (Rao, Leo, Bernardin, & Unverzagt, 1991). Cognitive impairment associated with MS may be found early in the disease course (Deloire et al., 2006), but occurs with increased frequency and severity in progressive MS (Planche, Gibelin, Cregut, Pereira, & Clavelou, 2016). In one study, the prevalence of cognitive dysfunction was shown to increase from 25% to 56% over a 10‐year interval. At baseline, patients had deficits in tasks of abstract reasoning, verbal memory, and linguistic processes, and after 10 years additional deficits in tasks of attention, and short‐term spatial memory were frequently seen (Amato, Ponziani, Siracusa, & Sorbi, 2001). In a population‐based study, 86% of SPMS and 74% of PPMS patients had significant cognitive impairment (Planche et al., 2016), and a study of decline in cognitive function showed that approximately 45% of patients had signs of cognitive impairment four years after their first MS symptom (Jonsson et al., 2006). Studies of cognition in patients with different disease courses show that progressive MS differ from RRMS (Drake, Carra, Allegri, & Luetic, 2006; Huijbregts, Kalkers, de Sonneville, de Groot, & Polman, 2006; Huijbregts et al., 2004; Ruet, Deloire, Charre‐Morin, Hamel, & Brochet, 2013), and other studies show that information processing speed is the primary cognitive deficit in MS regardless of the disease course (Rao et al., 1991; Van Schependom et al., 2014). Quality of life in MS may be affected by any of the symptoms stemming from the damage to the central nervous system or depression, anxiety, fatigue, mood disorder, cognition, vocational status, personality, and behavioral changes (Benedict et al., 2005; Janardhan & Bakshi, 2002).

The aim of this study was to assess to which degree quality of life correlates with commonly used measurements used in clinical trials involving MS patients including how it correlates with cognitive function. Thus, the overall aim was to provide data helpful in identifying the best suited outcome measures in clinical trials involving MS patients. To the best of our knowledge, there is no other paper that describes the relationship between 9‐Hole Peg Test (9HPT), Timed 25‐Foot Walk (T25FW) test, and quality of life in MS.

## 2 METHODS

This study is a retrospective analysis of data obtained from a double‐blind, placebo‐controlled, randomized study of 24 weeks of treatment with recombinant erythropoietin (EPO) in patients with SPMS and PPMS (Schreiber et al., 2016). Ethical approval was obtained from the local Danish Ethical Committee and all patients gave informed consent. At baseline, scores for Expanded Disability Status Scale (EDSS), T25FW, 9HPT, Paced Auditory Serial Addition Test (PASAT), Symbol Digit Modalities test (SDMT), and the Trail Making Test‐B (TRAIL‐B) were obtained. The SDMT was done orally to compensate for poor hand functioning. In addition, the patients answered the Short Form 36 (SF‐36) quality of life questionnaire.

### 2.1 Patients

Patients were included from 2 February 2010 to 31 May 2013. Inclusion criteria were as follows: a diagnosis of either PPMS or SPMS according to the revised McDonald criteria from 2005 (Polman et al., 2005); age between 19–60 years; EDSS of 4–6.5; and clinical progression without relapses of at least 0.5 points on the EDSS within the last 2 years. Main exclusion criteria were as follows: treatment with corticosteroids; interferon‐beta; glatiramer acetate within the last 30 days; or immunosuppressive treatment within the last 6 months prior to enrolment. MRI had to fulfill the Barkhof criteria for MS (Barkhof et al., 2003).

A total of 52 patients were included in the study: 18 had PPMS and 34 had SPMS. The study population consisted of 27 female and 25 male patients with a median age of 51 years (interquartile range [IQR] 47–57 years), median disease duration of 14 years (IQR 10–23), and a median duration of progression of 6 years (IQR 4–10). Demographics for the subdivided group are shown in Table 1.

**Table 1:** Demographics

	SPMS Median (IQR)	PPMS Median (IQR)
Age	50.5 (44.3–55.3)	52.5 (49.8–57.3)
Sex	17 men; 17 woman	8 men; 10 woman
Disease duration	11.0 (7.8–17.0)	10.0 (5.0–15.3)
Disease progression	5.0 (3.0–8.3)	10.0 (5.0–15.3)

### 2.2 Assessments

To assess different aspects of physical and cognitive function in MS several tests can be used. The most common clinical outcome measure is the EDSS(Kurtzke, 1983). It has its emphasis on ambulation at scores between 3 and 7, which comprises the majority of patients with progressive MS, and is insensitive to cognitive dysfunction and in this part of the scale also to upper extremity dysfunction.

Another commonly used outcome measure is the Multiple Sclerosis Functional Composite (MSFC), which is a composite score consisting of one test of ambulation (T25FW test), one test of upper extremity function (9HPT), and one test of cognitive function (PASAT) (Cutter et al., 1999; Koch, Cutter, Stys, Yong, & Metz, 2013).

The T25FW test is a quantitative measure of ambulation and has been used in clinical MS research for many years (Cutter et al., 1999). The 9HPT measures manual dexterity and gives a quantitative measure of arm and hand function. It is performed for both the dominant and nondominant hand and has high interrater reliability (Fischer, Rudick, Cutter, & Reingold, 1999; Oxford Grice et al., 2003). The PASAT assesses auditory information processing speed, flexibility, and calculation ability. Single digits are presented orally at a rapid rate and the patient must add each new digit to the one immediately prior (Fischer et al., 1999; Morgen et al., 2006).The SDMT is a widely used test of processing speed (Giovannetti et al., 2016). In addition it requires attention and concentration. The SDMT has been suggested as a sentinel test for cognitive impairment in MS (Langdon et al., 2012; Strober et al., 2009; Van Schependom et al., 2014). Single digits are paired with abstract symbols in rows of nine. The abstract symbols are arranged pseudorandomly and the patient must either say or write the number that corresponds with each symbol. The SDMT can be completed within 5 min including instructions, practice, and testing (Langdon et al., 2012). It has been reported to have a sensitivity of 82% and a specificity of 60% with a positive predictive value of 71% and a negative predictive value of 73% (Parmenter, Weinstock‐Guttman, Garg, Munschauer, & Benedict, 2007).The Trail Making Test (TRAIL) is included in many neuropsychological test batteries. It provides information on visual search, scanning, processing speed, executive functions, and mental flexibility. The TRAIL test consists of two parts: Test A and B (TRAIL‐A and TRAIL‐B), where the latter requires the subject to connect encircled numbers and letters in ascending and alternating order (e.g., 1‐A‐2‐B‐3, etc.). The score represents the amount of time required to complete the task (Arango‐ Lasprilla et al., 2015; Tombaugh, 2004). TRAIL‐B is generally considered to be a test of executive function, specifically rapid cognitive set switching and divided attention (Correia et al., 2015). The SF‐36 questionnaire is a frequently used measure of self‐reported health‐related quality of life not specific for any disease, age, or treatment group (Gandek, Sinclair, Kosinski, & Ware, 2004) and it is a brief and comprehensive test that works well on group level (Ware & Sherbourne, 1992). It consists of eight subscales measuring different aspects of health. The eight subscales are 1) physical functioning; 2) role limitations because of physical health problems; 3) bodily pain; 4) general health perceptions; 5) social functioning; 6) role limitations because of emotional problems; 7) vitality (energy/fatigue); and 8) general mental health (psychological distress and psychological well‐being) (Ware & Sherbourne, 1992). From the eight subscales two synthetic composites can be calculated: the Physical Component Summary (PCS) and the Mental Component Summary (MCS) where the PCS stems from subscale 1–4 and MCS stems from subscale 5‐8 (Straudi et al., 2015; Ware & Sherbourne, 1992). The PCS and MCS were constructed to simplify and improve the analysis of health outcomes (Jones, Jones, & Miller, 2004). A previous study established that MSFC scores correlate with SF‐36 scores and provide information about quality of life independent of the EDSS scores (Miller, Rudick, Cutter, Baier, & Fischer, 2000). We hypothesized that this can be caused by the inclusion of a measure of cognitive impairment, the PASAT, in the MSFC. Physical impairment as measured by EDSS is found to be associated with most physical and mental health‐related quality of life scores (Barker‐Collo, 2006; Benito‐Leon, Morales, & Rivera‐Navarro, 2002; Hopman et al., 2007) and a moderate correlation between SF‐36 quality of life and physical disability has been found (Haupts et al., 2003). Contributors to quality of life in MS can be depression, fatigue, mood disorder, cognition, vocational status, personality, and behavioral changes (Benedict et al., 2005; Janardhan & Bakshi, 2002).

### 2.3 Statistical analysis

Data are presented as median with interquartile range (IQR). Correlation analyses for baseline values were done using nonparametric Spearman′s rank correlation coefficient. Although some variables followed a normal distribution, for the sake of comparability, we used a nonparametric method for all analyses. Due to the large number of comparisons we used a Bonferroni correction resulting in a threshold of *p *< .001 for identifying statistically significant correlations. All statistical analyses were performed in SPSS version 22.

## 3 RESULTS

**Figure 1:**
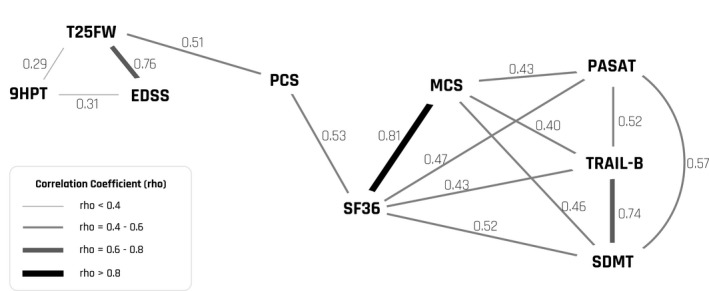
Graphical overview of all correlations between the measured variables

Baseline values for all variables are shown in Table 2. The SDMT and SF‐36 values were slightly higher in the 18 patients with PPMS compared to the 34 patients with SPMS (*p *= .024 and *p *= .045). When subdividing the combined group (Table 1) there was a longer progression duration for the PPMS patients (*p *= .005), but there were no other statistically significant differences. Figure 1 provides a graphical overview of all correlations between the measured variables. All measures of cognitive function correlated significantly with the results in the other cognitive tests (all *p *< .001), but did not correlate with the measures of physical impairment (Table 3). All measures of cognitive function correlated with the SF‐36 score (*p *= .001 or less), whereas we did not observe even nominally significant correlations between measures of physical impairment and the SF‐36 score (Table 3). Additionally, we investigated the relationship between the individual measures of physical impairment and cognitive function with the PCS and the MCS from the SF‐36 (Table 4). The SF‐36 score correlated strongly with the MCS and moderately with the PCS (rho = 0.81, *p *< .001, and rho = 0.53, *p *< .001), whereas the MCS and PCS did not correlate (rho = 0.024, *p *= .87). The PCS showed a significant correlation with the T25FW (Table 4: *p *< .001) but did not correlate with the EDSS score or the 9HPT score. The MCS correlated with all measures of cognitive function (*p *= .001 for PASAT; *p *= .003 for TRAIL‐B; *p *= .001 for SDMT) (Table 4). At last we conducted an exploratory analysis, in which we analyzed whether splitting 9HPT data into data for the dominant and the nondominant hand could yield additional information. This analysis revealed a nominally significant correlation between the 9HPT for the nondominant hand and the TRAIL‐B and SDMT (Table 5).

**Table 2:** Patient characteristics

	Median (IQR)
EDSS score	5.5 (4.5–6.0)
9HPT average both hands (s)	27.7 (24.3–33.4)
9HPT dominant hand (s)	27.1 (23.0–33.9)
9HPT nondominant hand (s)	30.2 (24.7–35.3)
T25FW (s)	9.1 (6.2–14.8)
PASAT (number correct of 60 possible)	50.0 (37.0–54.8)
SDMT (number correct in 90 s)	49.0 (40.5–57.3)
TRAIL‐B (sec to complete)	98.6 (65.3–118.1)
SF‐36	90.0 (79.1–101.7)
MCS	57.8 (47.9–62.2)
PCS	34.0 (29.0–39.5)

**Table 3:** Correlations between clinical scales and quality of life

	9HPT	T25FW	PASAT	TRAIL‐B	SDMT	SF‐36
EDSS	0.31 (*p *= .025)	0.76 (*p *< .001)^a^	−0.01 (*p *= .490)	0.002 (*p *= .989)	−0.13 (*p *= .347)	−0.01 (*p *= .836)
9HPT		0.29 (*p *= .040)	−0.1 (*p *= .484)	0.17 (*p *= .237)	−0.24 (*p *= .092)	−0.07 (*p *= .624)
T25FW			−0.11 (*p *= .427)	0.04 (*p *= .779)	−0.14 (*p *= .318)	−0.13 (*p *= .37)
PASAT				−0.52 (*p *=< .001)^a^	0.57 (*p *< .001)^a^	0.47 (*p *< .001)^a^
TRAIL‐B					−0.74 (*p *< .001)^a^	−0.43 (*p *= .001)
SDMT						0.52 (*p *< .001)^a^

a Statistically significant after Bonferroni correction (*p *< .001).

**Table 4:** Correlations between Mental Component Summary (MCS) and Physical Component Summary (PCS)

	MCS	PCS
EDSS	0.14 (*p *= .235)	−0.27 (*p *= .325)
9HPT	−0.06 (*p *= .658)	−0.07 (*p *= .642)
T25FW	0.14 (*p *= .308)	−0.51 (*p *< .001)^a^
PASAT	0.43 (*p *= .001)	0.17 (*p *= .239)
TRAIL‐B	−0.40 (*p *= .003)	−0.07 (*p *= .641)
SDMT	0.46 (*p *= .001)	0.12 (*p *= .406)

a Statistically significant after Bonferroni correction (*p *< .001).

**Table 5:** Correlations for dominant and nondominant 9HPT scores

	9HPT both hands	9HPT dominant	9HPT nondominant
EDSS	0.31 (*p *= .025)	0.29 (*p *= .040)	0.32 (*p *= .022)
T25FW	0.29 (*p *= .040)	0.24 (*p *= .084)	0.29 (*p *= .043)
PASAT	−0.1 (*p *= .484)	−0.15 (*p *= .305)	−0.08 (*p *= .577)
TRAIL‐B	0.17 (*p *= .237)	0.14 (*p *= .332)	0.31 (*p *= .027)
SDMT	−0.24 (*p *= .092)	−0.24 (*p *= .093)	−0.34 (*p *= .014)
SF‐36	−0.07 (*p *= .624)	−0.08 (*p *= .599)	−0.12 (*p *= .392)

## 4 DISCUSSION

The EDSS has been used as a clinical outcome measure for disability in MS research for many years. In the lower parts of the scale (0–3.0), the score depends on the presence of abnormalities in the neurological examination, at the middle part (3.5–7.0) almost exclusively on ambulation, and in the higher parts of the scale (7.5–9.5) on basic functions and ability to maintain activities of daily living. In order to overcome these shortcomings of the EDSS the MSFC, which combines the T25FW test as a measure of ambulation, the 9HPT as a measure of manual dexterity and the PASAT as a measure of cognitive function, is often used.

In this study, we evaluated the relationship between the individual parts of the MSFC and two additional measures of cognitive function. We did not aim at investigating the overall quality of life as compared to a control population, but rather to investigate the relationship between measures of cognitive function and motor function in relation to quality of life. In this context we found it relevant to investigate how the individual measurements correlated and thereby provide helpful evidence when choosing between different outcome measures in clinical trials and observational studies involving MS patients. We did not take into account how much quality of life was reduced in this population, but the inclusion of more disabled patients might have revealed a more clear relationship between physical function and quality of life which might be expected to correlate. We did not have measures of depression, anxiety, fatigue, mood disorder, vocational status, personality, or behavioral changes available in this study.

SF‐36 is a well‐recognized measure of quality of life. It is brief but yet comprehensive and works well on a group level. The high median value of SF‐36 in this study might be due to the fact that patients taking part in clinical trials are likely to be more resourceful, which may be a source of selection bias in this study. SF‐36 is a self‐reported measure of quality of life and patients with cognitive impairment and, for example, depression may have reduced insight which decreases the accuracy of self‐reported outcomes.

Our results are consistent with the strong bias toward ambulation impairment in the EDSS score. Surprisingly none of the physical measures correlated with the SF‐36 score, and only the T25FW test correlated moderately with the PCS from the SF‐36. This, most likely, reflects the relatively low number of patients with moderate ambulatory impairment included in this study. In contrast we found statistically significant correlation between two of the three cognitive measures and quality of life (Table 3).

We found a nominally significant correlation between scores for the nondominant 9HPT and the SDMT and TRAIL‐B scores, but the correlations were weak and should be analyzed in more detail in future studies before the significance of this finding can be assessed.

Cognitive impairment has been well documented to have negative impact on employment, social, and avocational activity (Benedict et al., 2002). All the cognitive tests used in this study correlated with the SF‐36 and the MCS, whereas there were no correlation between the cognitive tests and PCS. Furthermore, there was no correlation between the MCS and the PCS indicating that they reflect different aspects of quality of life, which ideally is what the two synthetic compound measures should do. The fact that the cognitive tests correlate with SF‐36 is most likely driven by the relatively strong correlation between the MCS and the SF‐36 score.

Correlation between the SDMT and PASAT has previously been reported in several studies (Brochet et al., 2008; Drake et al., 2010; Lopez‐Gongora, Querol, & Escartin, 2015; Nygaard et al., 2015). The highest correlation coefficient between the cognitive tests and the SF‐36 and the MCS was observed for the SDMT. The SDMT and the PASAT are both tests of processing speed (Fischer et al., 1999; Strober et al., 2009; Van Schependom et al., 2014). Both have their limitations but the SDMT has been suggested as the best choice for a reliable cognitive test in clinical research (Langdon et al., 2012; Strober et al., 2009; Van Schependom et al., 2014). It is simpler to administer than the PASAT, and has been found to be slightly more sensitive to MS cognitive impairment in RRMS patients (Lopez‐Gongora et al., 2015). Furthermore, the SDMT takes less time to complete, requires less expertise and experience of the assessor and, unlike the PASAT, it does not require special equipment for auditory presentation of stimuli. The SDMT and PASAT have equal psychometric validity (Drake et al., 2010). The SDMT can be performed either written or orally. The oral administration is recommended when testing MS patients to minimize confounding due to upper extremity weakness or ataxia (Benedict et al., 2002). A recent study did, indeed, report a correlation between the written SDMT and the 9HPT (Nygaard et al., 2015). Some authors have, however, found that speech is slow in MS patients, which could contribute to results when using neuropsychological tests such as both the oral SDMT and the PASAT that require rapid spoken response (Arnett, Smith, Barwick, Benedict, & Ahlstrom, 2008). SDMT performance can also be influenced by impaired visual acuity or visual scanning, whereas a potential confounder in interpretation of the PASAT is the calculation component that is associated to the premorbid calculation ability of the patient. The PASAT is perceived as challenging by most patients and unpleasant by some (Benedict et al., 2002). The SDMT and PASAT have both been found to significantly improve upon treatment with natalizumab in RRMS patients (Svenningsson et al., 2013). Since the SDMT is dependent on visual acuity a test such as a low‐contrast visual acuity test could help to eliminate this potential confounder and allow for a more detailed analysis (Baier et al., 2005; Balcer et al., 2003). Future studies could use a compound measure consisting of the 9HPT, T25FW test, and the oral SDMT in combination with a low‐contrast visual acuity test (MSFC‐4).

In conclusion, our study supports the need for including measures of cognitive function in clinical trials with progressive MS patients as cognitive function seems to be more closely associated with quality of life than physical impairment. Since information processing speed is a keystone in the cognitive problems of MS patients measures of this should be considered when choosing between cognitive tests.

## CONFLICT OF INTEREST

Dr. Højsgaard Chow reports non‐financial support from Genzyme, non‐financial support from Merck Serono, non‐financial support from TEVA, non‐financial support from Roche, non‐financial support from Biogen outside the submitted work. Dr. Schreiber reports grants from Roche Foundation for Anemia Research (RoFAR) grant award ID 9279576782 and Roche Denmark, commercial entity and Brdr. Rønje Holding, during the conduct of the study; other from Biogen Idec, personal fees from Genzyme‐ Sanofi, personal fees from Novartis, other from Merck Serono, other from Teva, outside the submitted work. Dr. Magyari reports personal fees from Consulting fee or honorarium from Roche, Novartis, Biogen Teva, grants from Novartis, Sanofi, Teva, Roche, Support for travel to meetings for the study or other purposes from Roche.Teva, grants pending from Biogen, personal fees from Payment for lectures from Novartis, outside the submitted work. Dr. Ammitzbøll reports non‐financial support from Teva, non‐financial support from Biogen Idec, non‐financial support from Genzyme, non‐financial support from Merck Serono, outside the submitted work. Dr. Börnsen reports non‐financial support for congress participation from Genzyme and Novartis, grants from Danish Mutiple Sclerosis Society, outside the submitted work. Dr. Romme Christensen reports non‐financial support from Novartis, outside the submitted work. Dr. Ratzer has had travel expenses reimbursed by Biogen Idec and Genzyme. Dr. Sorensen reports personal fees from Merck Serono, personal fees from TEVA, grants and personal fees from Novartis, grants and personal fees from Sanofi‐aventis/Genzyme, grants and personal fees from Biogen Idec, personal fees from GSK, personal fees from MedDay Pharmaceuticals, personal fees from Forward Pharma, outside the submitted work. Dr. Sellebjerg reports grants from Roche Foundation for Anemia Research, grants from Roche Denmark, grants from Brdr. Rønje Holding, during the conduct of the study; grants and personal fees from Biogen, personal fees from Merck, grants from EMD Serono, grants and personal fees from Novartis, personal fees from Teva, personal fees from Genzyme, outside the submitted work.
